# Matrix Metalloproteinase 3: A Promoting and Destabilizing Factor in the Pathogenesis of Disease and Cell Differentiation

**DOI:** 10.3389/fphys.2021.663978

**Published:** 2021-07-02

**Authors:** Jiangtao Wan, Guowei Zhang, Xin Li, Xianshuai Qiu, Jun Ouyang, Jingxing Dai, Shaoxiong Min

**Affiliations:** ^1^Spine Surgery, Zhujiang Hospital, Southern Medical University, Guangzhou, China; ^2^Guangdong Provincial Key Laboratory of Medical Biomechanics, Department of Anatomy, School of Basic Medical Sciences, Southern Medical University, Guangzhou, China

**Keywords:** matrix metalloproteinase 3, osteoarthritis, cell differentiation, stem cells, extracellular matrix

## Abstract

Cells must alter their expression profiles and morphological characteristics but also reshape the extracellular matrix (ECM) to fulfill their functions throughout their lifespan. Matrix metalloproteinase 3 (MMP-3) is a member of the matrix metalloproteinase (MMP) family, which can degrade multiple ECM components. MMP-3 can activate multiple pro-MMPs and thus initiates the MMP-mediated degradation reactions. In this review, we summarized the function of MMP-3 and discussed its effects on biological activities. From this point of view, we emphasized the positive and negative roles of MMP-3 in the pathogenesis of disease and cell differentiation, highlighting that MMP-3 is especially closely involved in the occurrence and development of osteoarthritis. Then, we discussed some pathways that were shown to regulate MMP-3. By writing this review, we hope to provide new topics of interest for researchers and attract more researchers to investigate MMP-3.

## Introduction

Matrix metalloproteinase-3 (MMP-3), also known as stromelysin-1, is a member of the matrix metalloproteinase family (MMPs). The function of this molecule depends on zinc ions in the internal environment. MMP-3 is composed of 475–478 amino acids in mammals, and its amino acid sequence is highly conserved among different species and genera, suggesting its potential for cross-species applications^1^. Like that of most MMPs, the structure of MMP-3 can be divided into four parts: a propeptide of approximately 80 amino acids, a metalloproteinase catalytic domain of approximately 170 amino acids, a ligating peptide of variable length (hinged region) and a heme protein domain of approximately 200 amino acids. MMP-3 can digest a variety of extracellular matrix (ECM) components, including matrix proteins, growth factors, proteases, surface receptors, and adhesion molecules. In particular, this enzyme can process a variety of pro-MMPs, and thus, the synthesis and activation of MMP-3 the first step in the MMP-mediated degradation process (Nagase et al., 2006; Pinto et al., 2006; Inge et al., 2012; Cui et al., 2017); furthermore, this molecule is associated with cellular fibrinolytic activity (Lijnen, 2002). Tissue inhibitor of metalloproteinase (TIMP) is a natural MMP inhibitor. As early as 2003, researchers proposed that the balance between MMPs and TIMPs is essential for the integrity of the ECM and that the proteolytic changes mediated by these molecules may lead to a variety of pathological states (Carine et al., 2003).

Matrix metalloproteinase 3 is an exocrine protein that is usually secreted through exocytosis and extracellular vesicles (EVs). Researchers extracted the exosomes of interleukin-1 beta (IL-1β)-treated synovial fibroblasts and detected MMP-3 in these exosomes, although the level was low (Kato et al., 2014). Another study found that adipose stem cells (ASC)-derived exosome-containing medium reduced tumor necrosis factor alpha (TNF-α)-induced osteocyte hypertrophy, and this effect was dependent on the abundance of MMPs and TIMPs in the exosomes (Niada et al., 2019). A previous study analyzed cytokines within ASC-derived exosomes to determine the cause of their chondrocyte-protective function. These researchers found high levels of TIMPs but barely any MMPs in the ASC-derived exosomes (Woo et al., 2020). Studies on the MMP-3 level in EVs are currently limited, and more efforts are needed to reveal the conditions in which MMP-3 can be secreted, taken up and function through EVs. MMP-3 can also be found in the nucleus and participate in the transcriptional process of chondrocytes separated from chondromas (Eguchi et al., 2008).

Current studies on MMP-3 mainly focus on tumor migration and central nervous system (CNS) development, such as axon growth and remodeling (Andries et al., 2017). In fact, as a stromal shaping agent, MMP-3 is widely involved in multiple cell biological processes, such as cell differentiation and inflammation. Given the extensive biological functions of MMP-3 and its involvement in a variety of diseases, this molecule can be applied as an indicator and therapeutic target of diseases. In the following sections, we summarize the biological functions and clinical applications of MMP-3 based on existing studies, hoping to provide new topics of interest for researchers and attract more researchers to investigate MMP-3.

## MMP-3 Extensively Participates in Cell Differentiation and Disease Progression

### Cell Differentiation

During differentiation, in addition to changes in the cell expression profile, an appropriate ECM environment is needed to sustain this process. In 1997, researchers proposed that MMP-3 expression is tissue-specific during morphogenesis of the rat maxillofacial region; that is, MMP-3 is expressed only in the region of mandibular ossification (Chin and Werb, 1997). Later, after analysis of the tissues of the lower jaw of patients with mandibular hypoplasia, researchers confirmed that MMP-3 genetic abnormalities can lead to mandibular hypoplasia. Based on *in vitro* experiments, BMAL1 defects induced by activating p65 phosphorylation were shown to enhance the transcription of MMP-3 and then inhibit osteogenic differentiation and promote osteoclast maturity, causing a decrease in bone mass, and eventually leading to mandibular hypoplasia (Jiajia et al., 2018). Researchers used antibody array analysis to study the cytokines secreted by stem cells derived from different tissues and concluded that MMP-3 expression levels differ between stem cells from exfoliated deciduous teeth (SHEDs) and bone marrow mesenchymal stem cells. These researchers also found that most stem cells constantly express MMP-3, suggesting that MMP-3 may play an important role in maintenance of stemness or differentiation (Yamada et al., 2019). Bone Morphogenetic Protein (BMP-4) can reduce the expression levels of both MMP-3 and MMP-13, reduce extracellular proteoglycan and collagen degradation, and finally inhibit C3H10T1/2 stem cell differentiation into adipocytes (Otto et al., 2007). Moreover, by observing and monitoring the changes in the levels of MMP-3 and TIMP-1 expressed by breast adipocytes during the lactation period, researchers found that MMPs, especially MMP-3, are involved in the proliferation and hypertrophy of adipocytes. They further confirmed this theory both *in vivo* and *in vitro* with 3T3-L1 cell lines (Alexander et al., 2001).

Noggin can promote the differentiation of mouse-derived embryonic stem cells into cardiomyocytes in a MMP-3-dependent manner (Hong et al., 2010). In the nervous system, transitional cells differentiated from adult neuroprogenitor cells (aNPCs) expressed increased levels of MMP-3 and MMP-9 under the effect of stromal cell-derived factor 1 and vascular endothelial growth factor. The above process can be interrupted by blocking the expression of MMP-3 or MMP-9 in aNPCs (Barkho et al., 2008). Axon inhibition is a major obstacle to nerve regeneration in adult mammals. Some researchers speculated that MMP-3 could abolish axon inhibition and then promote nerve regeneration by destroying the stromal layer and glial scar around neurons (Andries et al., 2017). Analyses of human embryogenesis and development further indicated that MMP-3 participates in the regulation of cell differentiation. RT-PCR, Western blots and enzyme activity analyses showed that the precursor and active forms of MMP-3 were expressed in villous trophoblasts during early pregnancy, differentiated invasive cytotrophoblasts and trophoblast SGHPL-4 cells (Husslein et al., 2009), suggesting that MMP-3 may play a role in early embryogenesis and germ layer differentiation.

### Disease Progression

**FIGURE 1:**
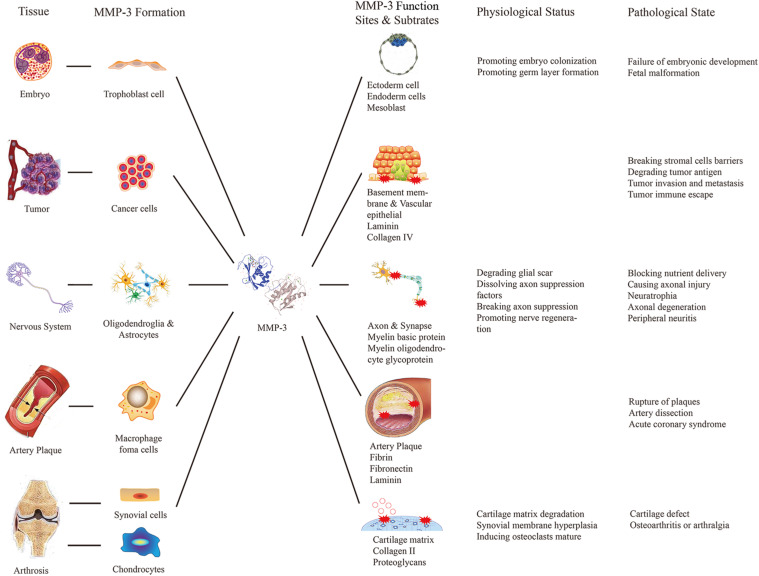
Relationship between diseases and MMP-3 biological functions. This figure listed the source, functional sites and the positive or negative effects of the MMP-3 in some particular tissues of the human body to explain the relationship between diseases and MMP-3 biological functions.

Matrix metalloproteinase 3 has many substrates, and thus, it is involved in the occurrence and development of various diseases (Figure 1). MMP-3 can degrade ECM, which is beneficial for tumor invasion and metastasis. Researchers compared MMP-3 mRNA levels between tumor cells and osteoblast cells in patients with osteosarcoma and concluded that osteosarcoma tumor cells express more MMP-3. Subsequent knockout experiments showed that MMP-3 knockout can reduce the migration of tumor cells, which confirmed that MMP-3 contributes to osteosarcoma invasion (Huang J. F. et al., 2016). Similar conclusions were reported by other researchers who inhibited the expression of MMP-3 with miR-134 (Chen et al., 2019). Moreover, other studies on ovarian cancer (Zheng et al., 2019) and anaplastic thyroid tumors (Ma et al., 2019) have reported the same effect of MMP-3.

Matrix metalloproteinase 3 is considered a common inflammatory factor that can promote inflammation in different parts of the body. In the nervous system, the typical pathological changes are extensive inflammation and axonal injury. Compared with that in the CNS, peripheral nerve injury, such as optic atrophy, macular degeneration numbness and hypesthesia, is more common (De Groef et al., 2015). Peripheral nerve injury caused by paclitaxel is thought to be related to excessive MMP-3 secretion (Nishida et al., 2008). However, the role of MMP-3 in the nervous system is complicated, and its ability to degrade ECM is considered to be a key factor in the disruption of the inhibitory CNS environment in the early stage of nerve regeneration (Andries et al., 2017). In the cardiovascular system, pathological changes caused by MMP-3 include endothelial injury and inflammatory cell accumulation. Studies have confirmed that the sustained expression of MMP-3 is closely related to atherosclerotic plaque rupture (Huang et al., 2017) because MMP-3 can interact with plasminogen and fibrinogen, showing antithrombotic activity. Some researchers have proposed that MMP-3 can be used to monitor plaque stability (Guizani et al., 2019) or as a target for preventing plaque rupture. Additionally, MMP-3 gene polymorphisms are closely related to the occurrence and development of cardiovascular diseases such as atherosclerosis and rheumatic heart disease (Huang X. Y. et al., 2016; Ghaffarpour et al., 2017; Hu et al., 2018). In musculoskeletal system, MMP-3 abnormalities can lead to pathological changes such as cartilage matrix degradation and synovial hyperplasia. High expression levels of MMP-1, MMP-2, MMP-3, MMP-8, MMP-9, and MMP-13 were found in the meniscus after inflammation or injury. The MMP-3 level in synovial fluid increased 30–40 times, while the level of TIMP-1 increased 10-fold within 24 h after meniscal injury (Lee et al., 2016).

## MMP-3 Is Closely Involved in the Occurrence and Development of Osteoarthritis

**FIGURE 2:**
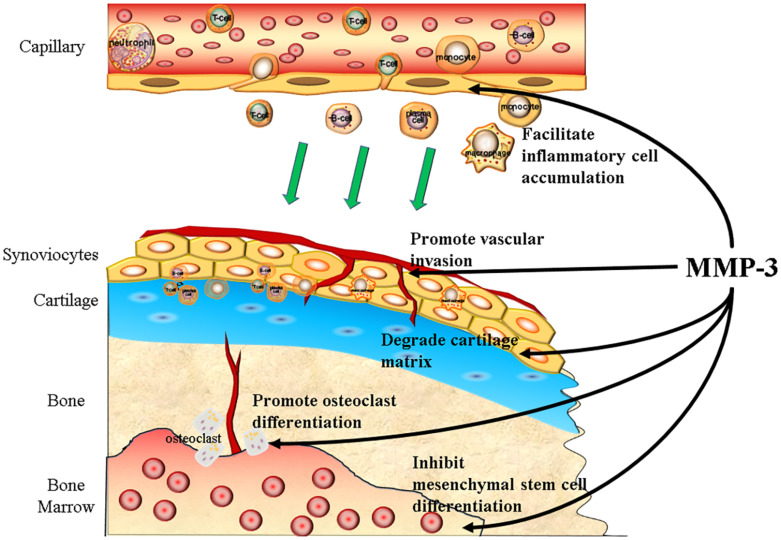
MMP-3 participates in the progression of OA. MMP-3 can promote the progression of OA both in preclinical and clinical stage. To be precisely, MMP-3 can facilitate the inflammatory cells accumulation, promote vascular invasion, degrade cartilage matrix, promote osteoclast differentiation, and inhibit mesenchymal stem cells differentiation.

Osteoarthritis (OA) is one of the most common degenerative diseases, and the prevalence of knee OA alone has reached 3.8%, affecting more than 250 million people worldwide (Cross et al., 2014). The typical pathological change in OA is cartilage degradation, and thus, MMP-3 participates in almost every step of the progression of OA (Figure 2).

At the preclinical stage of OA, MMP-3 levels increase in response to axial compression and degradation of cartilage matrix together with MMP-13 and ADAMTS-4 (Behrendt et al., 2016), while low mechanical load can reduce the release of MMP-3 and cartilage damage (He et al., 2020). Moreover, some particular MMP-3 genetic variation can lead to susceptibility to OA, according to a cross-sectional study including 431 females (Tong et al., 2017).

At the clinical stage of OA, MMP-3 can promote vessel growth into cartilage, facilitate inflammatory cell accumulation and inhibit mesenchymal stem cells from differentiating into chondrocytes. Vascular invasion is one of the signs of cartilage degeneration, and evidence has shown that MMP-3 participates in vascular formation (Chen et al., 2013). Normal cartilage does not have high levels of protease inhibitors (Franses et al., 2010), indicating it can be resistant to high MMP levels. The MMP-3 expression level in synovial cells is higher than that in cartilage, and slight angiogenesis is observed in the knee of OA patients (Koyama et al., 2020). Vascular invasion of bone marrow tissue into the subchondral plate is often observed in articular cartilage, and the cells around the invaded vessel express high levels of MMP-3 (Shibakawa et al., 2005). MMP-3 can disrupt the cell junctions between endothelial cells and degrade collagen within the ECM in the CNS, which facilitates the recruitment and accumulation of inflammatory cells (Behl et al., 2021). Compared with the CNS, articular cartilage lacks the protection of the blood-brain barrier; thus, inflammatory cells are more likely to infiltrate with the help of MMP-3. MMP-3 can inhibit osteogenic differentiation and promote osteoclast maturity. Osteoclasts can resorb non-calcified and calcified cartilage independently of acidification. MMPs and cysteine proteases mediate the resorption of calcified cartilage in OA (Lofvall et al., 2018). Loss of cartilage matrix not only causes chondrocyte hypertrophy and apoptosis (Kim et al., 2001; Hwang and Kim, 2015) but also inhibits mesenchymal stem cell or osteoprogenitor cell differentiation into chondrocytes (Toh et al., 2016; Yin et al., 2016).

Regarding the symptoms of OA, mild synovitis is very common. Studies have shown that MMP-3 is not directly involved in the progression of synovitis, but the products of decomposing cartilage matrix, such as cartilage oligomeric matrix protein and damage-associated molecule patterns, can recruit inflammatory cells, leading to synovitis (Foell et al., 2007; Huang et al., 2015; Haraden et al., 2019). In addition, MMP-3 is highly expressed in synovial cells during the inflammatory response (Liao et al., 2020), so MMP-3 promotes synovitis and synovitis promotes the expression of MMP-3, forming a vicious cycle in the progression of OA.

Fibrosis, which is excessive accumulation and abnormal composition of ECM, often follow synovitis, and its occurrence and regression are closely related to the balance of MMPS-TIMPs (Ko et al., 2019; Chen et al., 2021). Studies have found that MMP-3 is highly expressed in proliferative synovial tissues (Liao et al., 2020), and with the progression of fibrosis, the expression level of MMP-3 increases constantly, while the expression level of TIMP-1 decreases, leading to fibrosis regression (Hattori et al., 2017). The expression of MMP-3 only increases in the early stage of RA, a disease characterized by synovial hyperplasia and fibrosis, but a high expression of MMP-3 is maintained throughout the course of OA; this may be one of the reasons that the pathological changes that occur in OA and RA are different.

Pain is the main symptom of OA that seriously affects patients’ quality of life. Although MMP-3 has been reported to be involved in the development of peripheral neuropathy, researchers believe that MMP-3 is not directly related to pain in patients with OA (Fukushima et al., 2017). There is evidence that the effect of MSCs in the treatment of acute arthritis pain is not associated with specific changes in the synovial inflammatory cell population but instead is associated with the regulation of ADAMTS4, ADAMTS5, MMP3, and MMP9 expression in the synovial membrane after injury (Shu et al., 2020). However, no significant changes in MMP-3 levels have been observed in clinical studies on hyaluronic acid for the treatment of OA pain (Henrotin et al., 2019). Due to the lack of nerves in cartilage, patients do not experience significant pain in the early stage of OA. It has been confirmed that with the development of OA in cartilage, the subchondral fat pad (Heilmeier et al., 2020), subchondral bone and other structures are destroyed, resulting in the exposure or compression of nerve endings and thus intense pain. MMP-3 has been widely proven to be one of the major cytokines that damage the abovementioned structures (Belluzzi et al., 2019). Whether MMP-3 is directly related to pain in OA and whether it can be used as a therapeutic target for pain relief remain to be further studied.

## Mechanism of Regulating MMP-3 Expression and Secretion in OA

The mechanism regulating the expression and secretion of MMP-3 in OA has been investigated for a long time. In this section, we show that MMP-3 is predominantly regulated by the inflammatory factors TNF-α and IL-1β in some classic pathways.

Interferon regulatory factor 5 (IRF5), TNF-α and MMP-3 expression levels were increased in cartilage cells from patients with OA. Further research confirmed that IRF5 can increase the inflammatory factors within the culture system in a dose-dependent feedback regulatory mechanism *in vitro* (Lin et al., 2018). Both TNF-α and IL-1β can promote the synthesis and secretion of MMP-3 in preadipocytes (Gao and Bing, 2011). The expression of Kruppel-like factor 15 (KLF15) in chondrocytes was significantly reduced in patients with OA, while TNF-α treatment reduced p53-mediated KLF15 expression in human chondrocytes. Later studies confirmed that KLF15 can bind to the promoter of the MMP-3 gene and reduce TNF-α-induced MMP-3 expression at the transcriptional level (Yishuo et al., 2018).

### Nuclear Factor Kappa B Pathway

Nuclear factor kappa B (NF-κB) enhancer binding protein is a nuclear transcriptional activator that binds to enhancer elements in many different cell types and can be activated by a variety of pathogenic stimuli, such as stress, cytokines, free radicals, heavy metals, ultraviolet radiation, oxidized LDL, and bacterial or viral antigens (Choi et al., 2019). As early as 2002, some researchers proved that MMP-1 and MMP-3 were constantly expressed in human macrophages, and such expression was dependent on the activation of NF-κB. By transfecting inhibitory NF-κB subunits in cholesterol-induced rabbit foam cells, these scientists succeeded in reducing the expression levels of MMP-1/3 (Chase et al., 2002). Later, researchers verified that NF-κB could interact with the promoter of the MMP-3 gene through chromatin immunoprecipitation (Souslova et al., 2010). Activation of the NF-κB signaling pathway was found to be different between synovial cells and articular chondrocytes of OA patients, and the higher the level of RelA in the nucleus, the more MMP-3 expression was found in the cells (Ahmed et al., 2018).

### Mitogen-Activated Protein Kinase Pathway

The Mitogen-activated protein kinase (MAPK) pathway has three levels of signaling: MAPK, MAPK kinase (MEK or MKK), and the kinase of MAPK kinase (MEKK or MKKK). These three kinases can be activated in turn and together regulate many important physiological/pathological effects, such as cell growth, differentiation, stress and inflammatory responses. Some studies have confirmed that the MAPK pathway is involved in MMP-3 regulation in OA. By inhibiting MKK4 phosphorylation, miR-145 negatively regulates TNF-α-mediated JNK and p38 activation, as well as the nuclear accumulation of p-c-Jun and p-ATF2, ultimately leading to alterations in gene transcription (Hu et al., 2017). The levels of CX3CL1 in the synovium and serum are correlated with the incidence of OA. Further study proved that CX3CL1 increases MMP-3 levels by activating c-Raf, MEK, and ERK after binding with CX3CR1 (Hou et al., 2017).

### PI3K/Akt Pathway

The PI3K/Akt pathway appears to positively and negatively regulate MMP-3 expression. In the field of pharmacological research, oroxylin A was reported to reduce the upregulation of MMP-3 and MMP-13 expression by inhibiting the IL-1β-induced activation of ERK1/2 and the PI3K/Akt signaling pathway (Zhang et al., 2021). Scoparone can prevent IL-1β from promoting inflammation by suppressing Akt phosphorylation and promoting NF-κB p65 phosphorylation, thus reducing MMP-3 expression during the progression of OA (Lu et al., 2018). Another study suggested that moderate-intensity exercise can inhibit the inflammatory response of primary chondrocytes induced by IL-1β by increasing the level of 15-hydroxyeicosapetraenoic acid (15-HETE) in synovial fluid. The researchers found that 15-HETE could increase p-Akt levels and decrease the expression of MMP-3 and MMP-13 (Tian et al., 2021).

**FIGURE 3:**
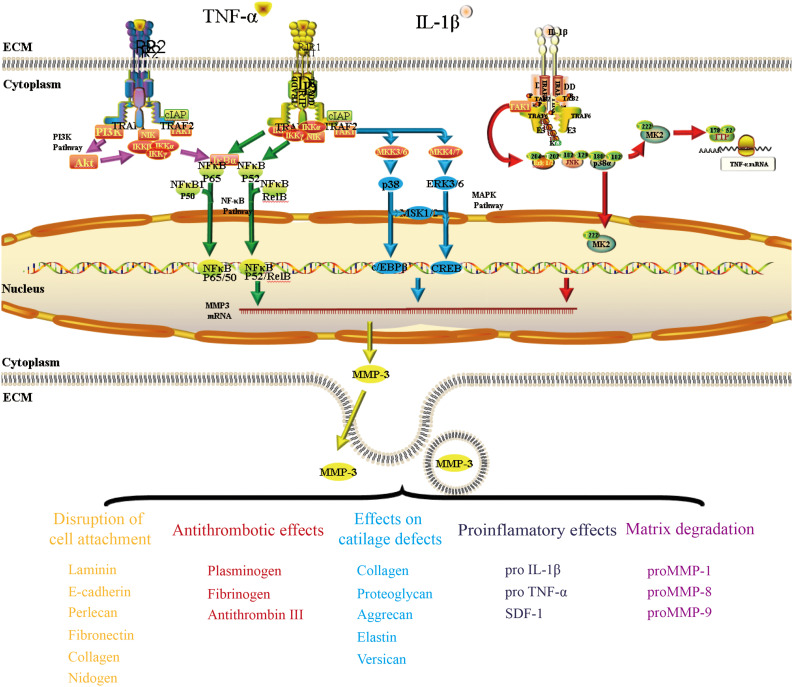
The mechanism by which TNF-α and IL-1β regulate MMP-3 expression in OA. This figure presented three pathways (NF-κb pathway, MAPK pathway, and PI3K/Akt pathway) that have been mentioned a lot when discussing the regulation of MMP-3 expression. TNF-α can bind to its traditional receptor TNFRI or non-classic receptor TNFRII and pass the signal through both NF-κb pathway. The transcriptional factor NF-κb could bind to the promoter of MMP3 gene and regulate MMP-3 expression. The binding of TNF-α and its receptors could also activate the TAK1 and then pass the signal through MAPK pathway. IL-1β could bind to its receptor and regulate MMP-3 expression through PI3K/Akt pathway.

We described some pathways that are relevant to the regulatory mechanism of MMP-3 in OA (Figure 3). The complete mechanism is too complicated to be described in a short review, and further details of these pathways must be elucidated.

## Clinical Detection and Inhibitors of MMP-3

### MMP-3 Detection

In inflammatory diseases involving bone and cartilage, such as rheumatoid arthritis (RA), ankylosing spondylitis (AS), and OA, the generally high level of serum MMP-3 suggests its potential as a diagnostic marker. Serum cartilage oligomeric protein and serum MMP-3 could be used as screening indicators to distinguish patients with knee arthritis from healthy people based on cross-sectional studies (Tsvetoslav et al., 2018). Researchers also found that the MMP-3 mRNA level in the intervertebral synovial fluid of AS patients was higher than that of the healthy group (Liu et al., 2015). However, the expression level of MMP-3 also changes along with disease progression and remission, which suggests MMP-3 may be a marker of curative effects. Serum MMP-3 levels in patients with OA were significantly decreased after treatment with non-steroidal anti-inflammatory drugs (Efstathiou and Settas, 2017) and decreased significantly in RA patients treated with certolizumab pegol (Ling et al., 2017). Other studies in which RA patients were treated with methotrexate monotherapy (Kazuko et al., 2016) and AS patients were treated with etanercept (Dongyi et al., 2017) showed similar results to those described above. Based on this extensive evidence, researchers have confirmed that the MMP-3 level in serum and synovial fluid can be used as a marker in both preclinical and clinical situations.

The traditional method of detecting MMP-3 is individual enzyme-linked immunosorbent assays (ELISAs), which is inefficient and time-consuming. With the development of immunodetection methods, many new technologies have been exploited and used in MMP-3 detection. Bead-based immunosorbent assays are modifications of ELISAs in which the affinity peptide is linked with magnetic beads and coated on a platform. As the liquid sample flows across the platform, the target molecules are captured by the affinity peptides, and then, specific antibodies are added to detect the target molecules in a high-throughput manner (Adamcova and Simko, 2018). Reliable testing methods for MMP-3 based on this technology, such as the Human Luminex Screening Assay (10 analytes; Silverstein et al., 2017; Wyatt et al., 2019) and Myriad Human InflammationMAP^®^1.0 multiplex immunoassay (47 analytes; Haraden et al., 2019), have been developed. Biosensors based on metal-plasma resonance have extremely high sensitivity when detecting inflammatory factors; they are also a high-throughput method that can screen many samples at the same time (Huang et al., 2013), but the high cost limits their applications. Recently, fluorescent probe techniques were used to detect MMPs and ADAMTs *in situ*. Using fluorescently labeled peptides to bind MMPs and ADAMTs as probes after injection and then excite fluorescence *in situ* with a laser, researchers can observe MMP expression in arthrosis, not just serum and synovial fluid (Mohammadinejad et al., 2020). The improvement and development of MMP-3 detection has significantly promoted the clinical application of MMP-3. However, some researchers have noted that detection of full-length MMP-3 does not accurately reflect the progression of the disease. Probes targeting the active region of MMP-3 should be developed to accurately reflect the disease state by detecting the expression level and the activation degree of MMP-3 (Bernotiene et al., 2020). Therefore, further research in this field is needed.

### MMP-3 Inhibitors

Researchers mainly inhibit MMP-3 expression through two methods, direct and indirect inhibition. First, by inhibiting TNF-α, IL-1β and its receptor, researchers can block the expression of MMP-3. Drugs corresponding to these mechanisms include anakinra, AMG 108 (a fully human monoclonal antibody to interleukin-1 receptor type 1), adalimumab, etanercept, and infliximab (Lespasio et al., 2017, 2018; Grassel and Muschter, 2020). Second, the transcription of MMP-3 can be directly blocked by miRNAs, including miRNA-155 (Korotkov et al., 2018) and miRNA-519d (Chengling et al., 2018), which have been experimentally tested. In addition, as a natural TIMP, TIMP-1 has an antagonistic function against MMP-3. However, due to its extensive and unpredictable side effects, this molecule cannot be applied in the clinic.

Because of their extensive anti-inflammatory effects, glucocorticoids are often used to control pain and relieve other symptoms in patients with RA, AS, and OA. Dexamethasone has been shown to inhibit the expression of endothelin in astrocytes, thereby reducing the synthesis of MMP-3 (Yutaka et al., 2017). Moreover, in traditional Chinese medicine, many botanicals have been proven to have multiple functions, such as anti-inflammatory, antioxidative, and antiapoptotic effects. Representative drugs include naringenin (Wang et al., 2017) or ginkgo biloba extracts (Zeng et al., 2015). *In vitro* experiments proved that these treatments can reduce the damage caused by MMP-3, but the specific mechanism is still unknown.

## Discussion

Matrix metalloproteinase 3 can show either positive or negative effects depending on its expression level and tissue specificity: the positive effects of MMP-3 play an important role in growth and development, especially the formation of some subtle structures, such as the early germ layer and maxillofacial bone. In adults, MMP-3 has been confirmed to promote axonal growth, cartilage ossification and adipose differentiation. The negative effects of MMP-3 are mostly related to the development of diseases. In the CNS, this molecule affects neurotrophy, causing axon damage, and neurodegeneration. In the cardiovascular system, MMP-3 aggravates endothelial injury and promotes plaque shedding. In musculoskeletal system, this enzyme degrades the matrix of articular cartilage and destroys the original structure of joints, losing its ability to protect against stress. However, due to its large number of substrates and the broad expression of natural inhibitors, it is difficult to separately discuss the relationship between diseases and the biological functions of MMP-3. Therefore, this review summarized the relationship between MMP-3 and diseases, especially OA, based on the existing evidence.

Current studies generally have a standard method of researching MMP-3. First, these studies explore how gene defects or the pathological internal environment affects MMP-3 expression. Second, researchers prove that MMP-3 expression is closely related to disease development and stop after these findings instead of investigating the MMP-3 interactions with specific cell components in physiological or pathological processes. MMP-3 has a variety of functions and causes obvious side effects when it is used as a therapeutic target. Elucidating the MMP cascade reaction initiated by MMP-3 or studying the reaction products of MMP-3 and its substrates can help us better understand the mechanism of action of MMP-3 so that we can use MMP-3 for clinical applications as soon as possible.

Matrix metalloproteinase 3 plays an important role in cell differentiation, especially in cell morphology and the ECM reshaping process. However, few studies have examined MMP-3 and stem cell-based tissue engineering. To date, most protocols for inducing stem cell differentiation include activating the inflammatory pathway, which can disrupt the homeostasis of stem cells and trigger the differentiation process. MMP-3 has both proinflammatory and anti-inflammatory effects. This molecule may be considered a target for regulating the differentiation of stem cells, as we did when researching OA and other inflammatory diseases related to MMP-3. In this review, we summarized the biofunctions of MMP-3 and its roles in cell differentiation and a variety of diseases, especially OA, hoping to draw more attention to MMP-3 and promote the study of its mechanisms.

## Author Contributions

JW, GZ, XL, XQ, JO, JD, and SM participated in the conception and writing of the manuscript. JD and SM reviewed and suggested modifications to the content. JD designed the structure of review manuscript. All authors contributed to the article and approved the submitted version.

## Conflict of Interest

The authors declare that the research was conducted in the absence of any commercial or financial relationships that could be construed as a potential conflict of interest.

## Footnotes

1 www.brenda-enzymes.org
